# Carcass characteristics, chemical composition, physicochemical, texture, and sensory properties of meat from White Kołuda® geese

**DOI:** 10.1016/j.psj.2026.106983

**Published:** 2026-04-20

**Authors:** Dariusz Kokoszyński, Dorota Cygan-Szczegielniak, Arkadiusz Nędzarek, Hanna Jankowiak

**Affiliations:** a Department of Animal Breeding and Nutrition, Bydgoszcz University of Science and Technology, 85-084 Bydgoszcz, Poland; b Department of Biology and Animal Environment, Bydgoszcz University of Science and Technology, 85-084 Bydgoszcz, Poland; c Department of Aquatic Bioengineering and Aquaculture, West Pomeranian University of Technology in Szczecin, 71550 Szczecin, Poland

**Keywords:** Goose, Gender, Type of muscle, Carcass composition, Meat quality

## Abstract

This study aimed to determine the effects of muscle type and gender on the proximate composition, concentrations of selected minerals, physicochemical and sensory properties of meat, as well as on carcass and edible offal characteristics in White Kołuda**®** geese. The research material consisted of 32 eviscerated carcasses and edible offal of the White Kołuda**®** goose breed at the age of 15 wk. Male White Kołuda**®** geese had a higher carcass weight (4475.2 g) compared to females (3955.1 g). The carcasses of males had a higher (*P* < 0.05) percentage of neck and wings and a lower percentage of skin with subcutaneous fat (*P* = 0.024), and higher weight of the heart, liver, and feet than females. A higher intramuscular fat content and less protein in the breast muscles, and more collagen in the leg muscles of males than females, were found, as well as a higher intramuscular fat and collagen content in the leg muscles than in the breast muscles, and a significantly higher protein content in the breast muscles. In addition, higher phosphorus and potassium content was found in the breast and leg muscles of females compared to males, and significantly higher phosphorus, iron, and copper content and lower sodium, zinc, manganese, and chromium concentration in the breast muscles than in the leg muscles. The male breast and leg muscles had significantly higher thermal loss and yellowness compared to the muscles of female geese. In addition, higher electrical conductivity, redness, and lower pH and lightness were found in the breast muscles than in the leg muscles. Male geese had significantly worse tenderness, hardness, aroma intensity, and greater springiness, chewiness, and gumminess of breast muscle compared to female muscles. Breast muscles had lower aroma intensity and desirability, tastiness intensity, and juiciness than leg muscles.

## Introduction

Goose meat production in 2024 amounted to 5,406,833 tons. This accounted for 3.7% of global poultry meat production. As in previous years, China dominated goose meat production, accounting for 97.8% (5,278,750 tons) of total production. In Europe, Poland and Hungary have alternated as the leaders in goose meat production in recent years (FAOSTAT, 2024). The current annual production of goose meat in Poland is approximately 20,000 tons, of which about 70% is exported, mostly to Germany (Lewko et al., 2023; Wereńska et al., 2026).

In Poland, approximately 90% of goose meat production comes from young commercial W31 hybrids (a hybrid of a male from the W33 paternal line and a female from the W-11 maternal line) of the White Kołuda® goose breed. A small percentage of meat is obtained from regional geese, geese from the W11 and W33 lines, including geese after reproductive use, and Tapphorn and Eskildsen meat line geese from Germany and their hybrids, as well as Nadwarciańska geese (Buzała et al., 2014; Kokoszyński et al., 2022; Dobrzyńska et al., 2025).

Poland specializes in the production of young Polish oat geese, kept until 17 or even 24 weeks of age and fed only whole oats during the last three weeks of their lives. The slaughter of young geese aged 8-12 wk is of little significance (Bielińska and Badowski, 2007; Buzała et al., 2014; Gumułka and Połtowicz, 2020). According to data from the National Poultry Council (KRD), in 2024, the average body weight of commercial geese aged 17 wk covered by the assessment (total 139 flocks, an average of 5.885 birds per flock) was 6.47 kg, FCR (feed conversion ratio) for concentrated feed 5.29 kg/kg BW gain, total mortality during rearing and fattening with oats 4.4% (Wencek et al., 2025).

The White Kołuda® goose breed is based on the genotype of Italian geese purchased for Poland from Denmark in 1962. Significant improvements in reproductive and meat performance as a result of several decades of breeding work at the Kołuda Wielka breeding farm near Inowrocław (Poland) led to the White Kołuda® goose being recognized as a separate Polish goose breed in September 2012. White Kołuda® geese are characterized by high egg production (50-60 eggs per year), egg fertilization and hatchability, and very good suitability for the production of young Polish oat geese (Korytkowski, 2022).

In recent years, White Kołuda® oat geese have been the subject of numerous studies, including those on slaughter traits and meat quality parameters (Bielińska et al., 2018; Biesek et al., 2020; Wołoszyn et al., 2020; Lewko et al., 2023). A review of the results of studies on growth performance, slaughter traits, and meat and fat quality in Polish oat geese was conducted by Buzała et al. (2014) and Nowicka and Przybylski (2018), among others. Scientific research on carcass characteristics and meat quality has recently been conducted, among others, in China (Huang et al., 2023; Chaiwang et al., 2026), Egypt (Geldenhuys et al., 2013; Geldenhuys et al., 2015), Czechia (Uhliřová i Tůmová, 2014; Uhliřová et al., 2018), Turkiye (Oz and Celik, 2015; Boz et al., 2019; Akbaș et al., 2020), and Lithuania (Rozmaitė et al., 2022).

Goose meat is not very popular in Poland, mainly due to its high price and the lack of tradition of eating goose meat. Meanwhile, goose meat is characterized by excellent taste, high dry matter, protein, and vitamin content, low fat content, and favorable fatty acid composition (Bielińska and Badowski, 2007; Razmaitė et al., 2022; Huang et al., 2023; Chaiwang et al., 2026). Compared to broiler chicken meat, goose meat contains more protein (22.8% vs. 21.4%), fat (7.1% vs. 3.1%), minerals (1.1% vs. 1.0%), and less water (68.3% vs. 75.5%). Goose meat lipids contain more monounsaturated and polyunsaturated fatty acids and more cholesterol (FoodData Central, 2026). Goose meat has a desirable ratio of n-6 to n-3 fatty acids. It surpasses chicken meat in terms of B vitamin content, with the exception of vitamin B_3_. 100 g of goose meat contains more minerals, including iron, copper, phosphorus, and potassium, than broiler chicken meat (FoodData Central, 2026).

Sex is one of the factors that influence slaughter traits and meat quality parameters. Studies have shown that male geese have higher body weight, carcass weight, giblets weight, breast and leg muscle mass, as well as higher fat mass (Murawska, 2013; Geldenhuys et al., 2013; Chaiwang et al., 2026). In addition, sex-related differences had a significant impact on the proximate composition, physicochemical parameters of meat (pH, drip loss, cooking loss, color attributes L*, a*, b*), tenderness, and flavor of meat (Uhlirová et al., 2018; Chaiwang et al., 2026).

Another factor affecting meat quality is muscle tissue type. Previous studies have found, among other things, that muscle type has a significant impact on meat microstructure characteristics, proximate composition, and physicochemical properties (Gumułka et al., 2009; Geldenhuys et al., 2013; Razmaitė et al., 2022).

Relatively few scientific studies on the texture characteristics of goose meat (Gornowicz et al., 2015; Wołoszyn et al., 2020; Kokoszyński et al., 2022; Razmaitė et al., 2022) and the mineral content in breast and leg muscles (Geldenhuys et al., 2015; Nowicka et al., 2018), as well as the lack of information on the electrical conductivity (EC_24_) and rheological properties (sum of elastic moduli, sum of viscous moduli) of White Kołuda® goose meat, were the reasons for conducting this research.

Electrical conductivity is a relatively little-known parameter of meat quality. Studies (Łyczyński et al., 2009) have demonstrated significant correlations between electrical conductivity and thermal drip, free water content, juiciness, water content and meat pH, all important characteristics for meat processing.

Assessing the texture and rheological properties of meat is one of the elements characterizing its technological and consumer quality. This analysis is particularly important in the case of goose meat, which is characterized by a different muscle structure resulting from differences in muscle fiber size and a relatively high proportion of connective tissue compared to other poultry species (Balowski et al., 2015). Rheological parameters describing viscoelastic materials such as meat reflect its ability to deform, resulting from the organization of muscle fibers and the proportion of connective tissue (Tornberg, 2005). It has been shown that the rheological properties of meat are closely related, among other things, to the content and structure of collagen, which influences, among other things, the quality of the meat on the hardness, elasticity, and rheological properties of this raw material (Purslow, 2005; Nishimura, 2010).

This study aimed to evaluate the carcass characteristics and meat quality of White Kołuda® geese, taking into account the influence of sex and/or muscle type. Breast and thigh muscle samples were taken from each carcass after dissection and used to determine proximate composition, selected minerals, physicochemical properties (pH_24_, EC_24_, thermal loss, L*, a*, and b*), sensory characteristics (tenderness, juiciness, aroma, and tastiness intensity and desirability), and samples of *m. pectoralis major* were collected for texture and rheological characteristics. The research hypothesis assumes that there may be differences between male and female White Kołuda® geese in terms of carcass composition, physicochemical and sensory characteristics of meat, and the impact of muscle type on the meat quality parameters studied.

## Materials and methods

### Materials

The research material consisted of 32 eviscerated carcasses with necks of the White Kołuda® breed and edible offal, i.e., hearts, gizzards (without digesta and creatine epithelium), livers (without gallbladders), and feet. The eviscerated carcasses and edible offal were obtained from 16 male and 16 female White Kołuda® geese aged 15 wk.

### Bird and rearing conditions

According to information provided by the breeder (White Kołuda Experimental Station of the National Research Institute of Animal Production, Poland), the birds were kept under the same environmental conditions and fed the same diets prior to slaughter. Until 4 weeks of age, the geese were kept in a closed building with windows (brooding house), but without access to a paddock. From 5 to 12 wk of age, the birds were kept in another building (with windows and access to a grass paddock). Until 4 weeks of age, the geese were fed a complete pelleted feed containing 20.0% crude protein (CP), 11.7 MJ metabolizable energy (ME), and 3.2% crude fiber (CF). From 5 to 8 wks of age, the geese were fed a pelleted feed mixture containing 18.5% CP and 11.5 MJ ME/kg feed and 4.5% CF, while from 9 to 12 wk of age, they were fed a fine feed mixture containing 14.5% CP, 11.0 MJ ME/kg feed, and 4.5% CF. During the rearing period (0–12 wk), 50 g (1 week of age) to a maximum of 270 g feed mixture (7–9 wk) was administered daily. The ingredients of the starter feed mixture used up to and including 4 wk of age were corn meal, extraction meal from genetically modified soybeans, extraction meal from rapeseed, wheat bran, calcium carbonate, monocalcium phosphate, soybean oil, sodium chloride, and sodium bicarbonate. The feed mixture given to geese from 5 to 8 wk of age also contained wheat and barley, while in the final rearing phase (9-12 wk of age), it also contained oats, wheat, and barley, in line with the composition of the starter mixture (0-4 weeks). In addition to feed mixtures, during the rearing period (from 1 to 12 wks of age), the goslings were also fed cut green forage, mainly consisting of grass, in amounts ranging from 20 g (in the 1 week of life, from 3 to 4 d of life) to 1500 g (12 wk of age). From 5 wk of age, the birds also had access to an open pasture on the farm. Additionally, from day 4 of life, during the rearing period, the birds were fed a mixture containing gravel (40%), feed chalk (10%), Avimix mineral mixture (40%), and MM-D mineral mixture (10%) in separate feeders. From 13 to 15 wk of age, i.e., for a period of three weeks, the geese were kept in the same closed building, but without access to a paddock (pasture) and fed voluntarily only whole oat grain. The oat grain contained 11.8 CP, 10.8 MJ ME/kg, and 8.9 CF. Each bird was fed 600 g of oats per day (at 13 wk of age), 500 g (at 14 wk), and 450 g (at 15 wk). During the fattening period, no green fodder was fed with the oats. Gravel was provided in separate feeders to improve the grinding of the oat grain. Throughout the entire rearing period (0-12 wk of age) and voluntary fattening with oat grain (from 13 to 15 wk of age), the geese had 24 h access to drinking water.

The temperature in the poultry house was 25-26 °C until the third day of life and was reduced to 18 °C from the fifth week of life. The building was heated with gas heaters. In addition, local heat and light sources in the form of infrared radiators were used up to and including the third week of life. Until the 3rd d of life, the temperature under the infrared radiator was 29-31 °C and was gradually reduced; in the 3rd week of rearing, it was 23-21 °C. The relative humidity in the poultry house was 65-70%. The geese were raised under veterinary supervision, adhering to biosecurity and goose welfare standards. At 14 days of age, the geese were vaccinated against Derzsy's disease. The geese were slaughtered and eviscerated at the Kołuda Wielka Experimental Station slaughterhouse of the National Research Institute of Animal Production (Poland). The Kołuda Wielka Experimental Station is authorized to directly sell goose carcasses and offal as part of its agricultural operations.

### Analysis of carcass composition

After purchase, the gutted carcasses and edible offal, cooled in a refrigerated cabinet, were transported by car in a refrigerated box at a temperature of 4 °C to the University laboratory. The transport time of the carcasses and edible offal to the laboratory was 50 min. Upon arrival at the laboratory, the carcasses and edible offal were weighed individually using a WLC 6/12/F1/R electronic scale (Radwag, Radom, Poland). Then, the whole carcasses with necks were dissected using a simplified method (Ziołecki and Doruchowski, 1989). During dissection (carcass cutting), the following parts of the carcasses were sequentially separated: abdominal fat, wings with skin, neck without skin, skin with subcutaneous fat from the entire carcass, skinless wings, breast muscles (fillet – *pectoralis major* muscle and tenderloin – *pectoralis minor* muscle from both sides of the breast), leg muscles (all thighs and drumsticks muscle from both legs), and carcass remains (trunk, leg bones). The carcass elements extracted during dissection were weighed individually on an electronic scale (WLC 6/12/F1/R), and then their percentage share in the cold carcass gutted with the neck was calculated.

### Proximate analysis

The water, protein, intramuscular fat, and collagen contents were determined according to PN-A-82109 (2010), using FoodScan™ 2 Meat Analyser (FoodScan, Hillerød, Denmark) with a Near Infrared Transmission (NIT) spectrometry, calibrated for an ANN (artificial neural network).

### Mineral determination

At the beginning, breast or thigh samples were freeze-dried. Freeze-drying of the samples was carried out in an Alpha 1–4 LD plus dryer (Christ, Osterode, Germany). The freeze-drying parameters were as follows: minus 50°C and a pressure of 0.63 atm. Freeze-drying time: 24 h, until a solid mass was obtained. Then, freeze-dried sample preparation involved high-pressure microwave digestion using a Speedwave Xpert system (Berghof, Germany). A freeze-dried goose meat sample weighing approximately 0.5 ± 0.01 g was dissolved in 6.0 mL of a mixture of ultra-pure (Merck, Germany) concentrated acids: nitric acid (V) (HNO_3_) and chloric (VII) (HClO_4_) acids mixed in a 5:1 ratio. The mixture was left at room temperature for 1 h. The samples were then dissolved according to the following program: step one, temperature increase to 170°C within 10 min and maintenance for 5 min, pressure 30 bar; step two, increase the temperature to 200°C within 3 min and maintain for 20 min, pressure 30 bar; step three, temperature 50°C, hold time 10 min, pressure 25 bar. After cooling to room temperature, the dissolved samples were diluted with Milli-Q water (18.2 MΩ) to a final volume of 25 mL. The metal concentrations were quantified using a Hitachi ZA3000 Series Polarized Zeeman Atomic Absorption Spectrometer (Hitachi High-Technologies Corporation, Tokyo, Japan). The concentrations of chrome (Cr), copper (Cu), iron (Fe), manganese (Mn), and zinc (Zn) were measured using graphite furnace atomic absorption spectroscopy (GFAAS). Whereas calcium (Ca), potassium (K), sodium (Na), and magnesium (Mg) were determined by flame atomic absorption spectroscopy (FAAS) in an air-acetylene flame. Phosphorus (P) was determined by a colorimetric method with ammonium molybdate and ascorbic acid as the reducing agent. Absorbance was measured at a wavelength of λ = 882 nm using a Hitachi U-2900 double-beam UV-VIS spectrophotometer (Tokyo, Japan). All markings were made in three repetitions.

Calibration curves were prepared using certified single-element standard solutions (1000 mg/L) from Scharlau (Barcelona, Spain) for Ca, Fe, K, Mg, and Na and from Merck (Germany) for Cu, Cr, Mn, P, and Zn. Calibration curves were established, with correlation coefficients (r) required to be at least 0.995.

Accuracy of the analytical procedure was assessed using the certified reference material Dogfish Liver NCR-DOLT-5 (National Research Council Canada). Elemental recoveries were 104% (for Ca), 93% (for Cr), 101% (for Cu), 103% (for Fe), 106% (for K), 99% (for Mg), 103% (for Mn), 114% (for Na), 107% (for P) and 98% (for Zn).

### Physicochemical analysis

pH measurements of breast and leg muscles (thigh) were performed with a multifunctional device, the CX-700 (Elmetron, Zabrze, Poland). This device was equipped with a knife electrode, which was inserted into a sample of breast or thigh meat during the measurement. In turn, the electrical conductivity (mS/cm) of the *pectoralis major* and thigh muscles was measured 24 h after slaughter using an LF-Star CPU conductivity meter (Ingenieurbüro R. Matthäus, Nobitz, Germany). During the EC24 measurement, with a precision of 0.01 mS/cm, the device's electrodes were placed in the pectoralis major or thigh muscle at an angle of 90° along the muscle fibers.

Thermal loss was determined according to the method described by Walczak (1959). Meat samples from breast or thigh muscles, weighing 20 *g* ± 2 g, were minced into small pieces, formed into balls, wrapped in absorbent gauze, and placed in a water bath at 85°C for 10 min. The samples were then removed from the water bath. They were cooled for 30 mins in a refrigerator at 4°C and then weighed again on a WPS 210C scale (Radwag, Radom, Poland). Losses during meat cooking were calculated based on the difference between the weight of the sample before and after heat treatment. The results of cooking losses were expressed as a percentage of the initial sample weight.

The color of meat was determined by the CIELab method on the inner surface of raw *pectoralis major* or thigh muscles. Color lightness (L*), redness (a*), and yellowness (b*) were determined. The color coordinates were measured with a Konica Minolta colorimeter (Konica Minolta, Japan) with a CR410 head, using the following measurement parameters: measurement area about 0.5 cm², measuring head slot 8 mm, illumination D65, observer 2°, calibration on the white tile according to *Y* = 86.1, *x* = 0.3188, *y* = 0.3362. The color parameters (L*, a*, b*) were measured 3 times (cranial, medial, caudal locations) for each sample *of m. pectoralis major* or thigh muscles. In total, 32 samples of pectoral muscle and 32 samples of thigh muscle were measured.

### Sensory analysis

The sensory properties were evaluated on heat-treated muscles. The sensory assessment determined tenderness, juiciness, aroma, and tastiness intensity and desirability of breast and thigh muscles from oat White Kołuda® geese aged 15 wk. Sensory evaluation of the meat was carried out in the laboratory of Bydgoszcz University of Science and Technology, Poland.

Heat treatment of breast (*m. pectoralis major*) or thigh muscle (without skin, bones, patella) samples (weighing 40 g each) was conducted in 0.6% NaCl. 200 mL of water was added per 100 g of meat. The samples were heated to a temperature of 80 °C in the geometric center of the sample. After thermal treatment, the samples were cooled to 60 °C, cut into 1.0 × 1.0 × 1.0 cm cubes, and subjected to sensory assessment (Krełowska-Kułas, 1993). The assessment was performed by a panel of 6 trained judges with previous long experience in sensory evaluation of meat according to a 5-point scale provided by Baryłko-Pikielna and Matuszewska (2009). Meat tenderness was determined based on the following scale of assessment: 1 point – very hard, 2 points – hard, 3 points – slightly tender, 4 points – tender, 5 points – very tender. Juiciness of meat was determined using the following scale: 1 point – clearly dry, 2 points – slightly dry, 3 points – weakly juicy, 4 points – juicy, 5 points – very juicy. The assessment scale for intensity of aroma and tastiness was as follows: 1 point – imperceptible, 2 points – perceptible, 3 points – weakly distinct, 4 points – distinct, 5 points – very distinct. Aroma and taste desirability was assessed using the scale as follows: 1 point – very undesirable, 2 points – undesirable, 3 points – neutral, 4 points – desirable, 5 points – very desirable.

### *Determination of texture and rheological properties* of breast muscles

For texture and rheological properties determinations, the breast muscles were heat-treated in their entirety. Each muscle was individually packaged in PE bags and heated in a water bath at 72°C until the temperature at the geometric center of the samples reached 70.2°C and then cooled to room temperature. The test samples were 22 mm thick slices cut from the heat-treated breast muscle, perpendicular to the muscle fibers. The samples were cut at 40% of the length of the pectoralis major muscle from the coracoid side.

The texture of the meat was analyzed using the TPA (Texture Profile Analysis) test and the Warner-Bratzler (WB) test (Bourne, 2002) with the Stable Micro Systems TA.XT plus strength tester (Stable Micro Systems, United Kingdom). In the TPA test, a traverse speed of 50 mm min-1 and a deformation limit of 80% (16 mm) were adopted; a 50 N load cell and a cylindrical measuring pin with a diameter of 0.62 cm were used. From the force-deformation curve, parameters such as hardness, cohesiveness, springiness, chewiness, and gumminess were calculated. In the Warner Bratzler (WB) test, a traverse speed of 50 mm/min was used; the samples were cut parallel to the muscle fibers using a ‘V’ slot blade, and the maximum cutting force and cutting work were determined. The TPA and WB tests were repeated 6 times for each sample.

The Instron 1140 device (Instron, U.S.A.) was used to analyze rheological properties using a relaxation test. A measuring pin with a diameter of 0.96 cm was used, a deformation limit of 10% was assumed, and stress changes were recorded for 90 seconds. The generalized Maxwell model consisting of a Hooke body connected in parallel with two Maxwell bodies (Lachowicz, 1992) was used to calculate the elasticity and viscosity modules.

The equation for this model is as follows:

$$\delta =\varepsilon \cdot \left[E_{0}\cdot \exp\left(\frac{-E_{1}\cdot t}{\mu_{1}}\right)+E_{2}\cdot \exp\left(\frac{-E_{2}\cdot t}{\mu_{2}}\right)\right]$$

where:

δ – tension (kPa)
ε – deformation
E_0_ – Hooke's elasticity module (kPa)
E_1_,E_2_ - moduli of elasticity of Hooke's bodies 1 and 2, respectively (kPa)
µ_1_, µ_2_ - viscosity modules 1 and 2 of Maxwell's body, respectively (kPa x s)
t - time

In order to interpret the results, the sum of the elastic moduli (E0 + E1 + E2) and the sum of the viscosity moduli (µ1 + µ2) were calculated. The relaxation test was repeated six times for each sample.

### Statistical analysis

Statistical analyses were performed for the data concerning carcass and meat quality of oat geese using SAS (SAS Institute Inc, 2014). Each bird constituted an individual experimental unit for all analyzed carcass and meat quality traits. The arithmetic mean and standard error of the mean (SEM) were calculated for each trait. The normality of distributions was assessed using the Shapiro–Wilk test, while the homogeneity of variances was evaluated using the Fisher and Levene tests. In most cases, the analyzed variables were characterized by approximately normal distributions. Although some variables showed minor deviations from normality, these were not substantial and did not affect the validity of the applied parametric methods. The comparative analysis of the collected data was performed in two stages. In the first stage, the effect of sex on carcass traits was evaluated using the Student’s t-test. In the second stage, a two-way analysis of variance (ANOVA) was applied to determine the effects of sex and muscle type on the analyzed meat quality traits. The following model was used: Y_ij_ = µ + a_i_ + b_j_ + (a x b)_ij_ + e_ijk_ - was applied for meat quality traits, where: µ - was marked as the overall mean of the trait, a_i_ - was marked as the effect of the i_th_ sex, b_j_ - was marked as the effect of the j_th_ type of muscle, (a x b)_ij_ - was marked as sex by type of muscle interaction, and e_ijk_ - was marked as random error. Differences between means (between males and females or between breast and thigh muscles) were evaluated using Tukey’s test at *P* < 0.05*.* For all carcass and meat quality traits examined in this study, the experimental unit was the individual bird.

## Results and discussion

The study showed that 15-week-old male White Kołuda® geese had a significantly (*P* < 0.001) higher carcass weight (4475.2 g) compared to females (3955.1 g) of the same age (Table 1). Other studies conducted on geese (Geldenhuys et al., 2013; Murawska, 2013; Uhliřová et al., 2018; Huang et al., 2023; Chaiwang et al., 2026) also found (*P* < 0.05) significantly higher carcass weight in males than in females. Chaiwang et al. (2026) indicate that the higher carcass weight of males is associated with faster growth rates and greater muscle accretion compared to females. In turn, Murawska (2013) indicates that the significantly higher carcass weight of male geese is associated with a significantly higher percentage of lean meat, some internal organs, and bones compared to female geese. Sexual dimorphism in carcass weight is likely due to different levels of sex hormones in males and females that determine their growth.

**Table 1:** Carcass characteristics of male and female White Kołuda® geese at 15 weeks of age.

Item	Carcass weight (g)	Breast muscle (%)	Leg muscle (%)	Abdominal fat (%)	Skin with subcutaneous fat (%)	Wings (%)	Neck without skin (%)	Carcass remainders (%)
Male	4475.24^a^ ± 286.03	15.56 ± 1.63	13.32 ± 1.61	5.54 ± 1.40	23.38^b^ ± 2.33	13.16^a^ ± 0.82	5.55^a^ ± 0.71	23.45 ± 2.53
Female	3955.08^b^ ± 244.86	15.15 ± 2.12	13.40 ± 0.89	5.48 ± 1.21	25.54^a^ ± 2.81	12.36^b^ ± 1.06	5.00^b^ ± 0.74	22.06 ± 1.35
Average	4215.16 ± 362.18	15.85 ± 1.83	13.36 ± 1.55	5.51 ± 1.21	24.46 ± 2.75	12.76 ± 0.96	5.28 ± 0.74	22.76 ± 2.02
SEM	64.10	0.32	0.27	0.21	0.49	0.18	0.13	0.36
*P* value	<0.001	0.376	0.890	0.893	0.024	0.017	0.033	0.051

Note: Values are expressed as means ± standard deviation, *n* = 16 for gender.

^a,b^*P* < 0.05 – means with different superscripts are statistically different between male and female.

The leg muscle is the sum of all the thigh and drumstick muscles from both legs.

SEM - standard error of the mean.

The carcasses of 15-wk-old males were characterized by a higher content of breast muscle (15.56%), abdominal fat (5.54%), wings with skin (13.16%), neck without skin (5.55%), and carcass residue (23.45%) compared to female carcasses (15.15%, 5.48%, 12.36%, 5.00%, and 22.06%, respectively). In turn, carcasses of 15-wk-old female geese had a higher content of leg muscle and skin with subcutaneous fat than carcasses of males. Significant differences between males and females were found in terms of the content of skin with subcutaneous fat (*P* = 0.024), wings (*P* = 0.017), and neck (*P* = 0.033) - Table 1. Compared to the geese studied, Biesek et al. (2020) found lower breast muscle content (14.42–14.59%), leg muscle content (10.78–12.00%), and abdominal fat content (4.73–5.13%) and higher skin with subcutaneous fat content (26.15–26.98%) in the carcasses of 17-wk-old White Kołuda® geese. In studies by Lewko et al. (2023) found a higher total content of breast and leg muscles in gutted carcasses (males - 29.28%, females 30.50%), skin with subcutaneous fat in males (26.06%) and a lower percentage of carcass residues (males – 20.97%, females – 21.40%) in 17-wk-old White Kołuda® geese carcasses compared to 15-wk-old White Kołuda® geese carcasses reared according to the same rearing and fattening recommendations in both studies. Chaiwang et al. (2026) found a significantly higher thigh muscle content in 16-wk-old male White Chinese and Grey Chinese geese, while Kowalczyk et al. (2013) found a significantly lower percentage of breast muscle in 17-wk-old male geese compared to females of the same age. In turn, Huang et al. (2023) in 10-wk-old Xingguo Gray geese, Uhliřová et al. (2018) in 8- and 16-wk-old Eskildsen Schwer and Czech Goose did not find significant differences in carcass composition between male and female geese.

These studies found a significantly higher percentage of heart (*P* < 0.001), liver (*P* = 0.010), and feet (*P* < 0.001) in 15-wk-old male White Kołuda® geese than in female geese of the same age. In another study, Murawska (2013) showed a significantly higher heart and gizzard weight in male White Kołuda® geese compared to females. In turn, Biesiada-Drzazga (2014) found a significantly higher foot weight in male geese than in female geese of the White Kołuda® breed from the W11 line.

Analysis of the results of basic chemical composition measurements (Table 3) showed a significantly higher intramuscular fat content (*P* = 0.014) in the breast muscles and collagen (*P* < 0.001) in the leg muscles of males than in females, as well as a significantly lower intramuscular fat content in the leg muscles of males than in females. In addition, a significantly (*P* < 0.001) lower protein content was found in the breast muscles of males than in females at 15 wk of age. Moreover, a significantly higher intramuscular fat content (*P* < 0.001) and collagen content (*P* < 0.001) were found in leg muscles than in breast muscles, and a significantly higher protein content in breast muscles (*P* < 0.001). The interaction between gender and muscle type was significant for intramuscular fat and protein content. The results of other studies vary in terms of the influence of gender and muscle type on proximate composition. Geldenhuys et al. (2013) found significantly higher fat content and lower moisture content in the meat of male Egyptian geese compared to female meat. In contrast, Uhliřová et al. (2018) report significantly higher protein content in the breast muscles of 8- and 16-week-old male Eskildsen Schwer and Czech Goose geese and no significant effect of sex on moisture, crude fat, and crude ash content. Chaiwang et al. (2026) show a significantly lower fat content in the breast and leg muscles of males compared to females of 16-week-old Chinese geese. In contrast, Rozmaitė et al. (2022) found no significant effect of sex on the proximate composition – dry matter, protein, fat, and ash contents in the muscles of 10-wk-old Lithuanian Vištinés geese.

Analysis of the results of the determination of the concentration of the tested mineral components (Table 4, Table 5) showed a significantly higher content of phosphorus (*P* = 0.001) and potassium (*P* = 0.008) and a lower content of zinc (*P* = 0.017) in the breast muscles and leg muscles of females compared to males. In addition, significantly higher phosphorus, iron, and copper content and lower sodium, zinc, manganese, and chromium concentrations were found in breast muscles than in leg muscles (*P* < 0.001 to 0.007). Another study (Oz and Celik, 2015) found higher phosphorus (P), sodium (Na), calcium (Ca), and magnesium (Mg) content, but lower potassium (K) concentration in the breast and leg muscles of geese compared to the results of this study. Compared to data from FoodData Central, 2026, 100 g of breast and leg muscles from 15-week-old geese contained less calcium (Ca), phosphorus (P), potassium (K), and sodium (Na), and more magnesium (Mg) and manganese (Mn). The concentration of iron (Fe) and magnesium (Cu) in the breast muscles of the tested 15-wk-old White Kołuda® geese was similar, while in the leg muscles it was lower than that given in FoodData Central, 2026. According to Lewis's (2019) recommendations, 100 g of raw breast muscle of the tested 15-week-old White Kołuda® geese provides 31.7; 0.3; 8.2; 17.5; 10.7; 33.3 and 1.0% of Nutrient Reference Values – Requirements (NRVs – R) for P, Ca, Mg, Fe, Zn, Cu, and Mn. In turn, 100 g of raw leg muscle provides 29.6, 0.3, 8.3, 10.9, 24.7, 13.3, and 1.3% of NRVs-R for P, Ca, Mg, Fe, Zn, Cu, and Mn. Gołuch-Koniuszy and Haraf (2018) report that consumption of 100 g of goose meat covers most of the requirement for Se (46.4–63.8%), followed by P, Zn, Cu, and Fe, and least of all Ca and Mn (below 2%) of RDI – Recommended Dietary Allowances.

Analysis of physicochemical characteristics (Table 6) showed that breast and leg muscles of 15-week-old male White Kołuda® geese had significantly higher thermal leakage and yellow color saturation compared to muscles of 15-wk-old female geese. These studies also found significantly higher electrical conductivity (EC_24_), redness (a*), and acidification (lower pH_24_), and lower lightness (L*) in breast muscles than in leg muscles. Significant interactions between gender and type of muscle were also confirmed for pH_24_ and EC_24_. The pH values obtained for breast muscles 24 h after slaughter (pH_24_) of 15-wk-old White Kołuda® geese in this study were similar to the pH_24_ of breast muscles (pH_24_ = 6.22–6.24) of 16-week-old White Kołuda® geese in the study by Biesek et al. (2020). In contrast, Kowalczyk et al. (2013) found lower pH_24_ values (male 5.90, female 5.96) in the breast muscles of 17-week-old White Kołuda® geese. Uhliřová et al. (2018) also report significantly higher acidification (lower pH_24_) of the breast muscles of 16-week-old Eskildsen Schwer and Czech Goose geese compared to the breast muscles of geese in our own studies. It should be noted that the higher pH_24_ of leg muscles than breast muscles was probably related to the lower glycogen content in leg muscles, resulting from greater physical activity of this part of the body before slaughter. The lower concentration of glycogen in the leg muscles than in the breast muscles resulted in lower lactic acid production and thus less acidification (higher pH) of the leg muscles compared to the breast muscles. The thermal loss (TL) values obtained in this study for breast and leg muscles were lower than in the study by Chaiwang et al. (2026) involving White and Gray Chinese geese, where the TL of the breast muscles of males was 38.40% and that of females was 40.40%, while that of the leg muscles was 35.78% and 38.49%, respectively. In turn, Gumułka et al. (2009) report similar TL of breast muscles (30.35%) and leg muscles (27.67%) in 17-wk-old White Kołuda® geese to the muscles of geese tested at 15 weeks of age. The significant differences in meat thermal loss between males and females of 15-wk-old White Kołuda® geese found in this study were probably due to differences in protein solubility and fat content (Dobrzyńska et al., 2025).

Another relatively poorly understood indicator of goose meat quality is electrical conductivity (EC). EC is commonly used as an indicator related to water-holding capacity and drip loss. Studies (Kokoszyński et al., 2022) have shown little variation between Cuban, Slovakian, and Landes geese in terms of EC_24_ of breast and leg muscles. Color, on the other hand, is considered an indicator of meat freshness and suitability for culinary and processing purposes. Geese are a species of bird with red meat, high iron content, and heme pigments. Studies by Kowalczyk et al. (2013) and Razmaitė et al. (2022) found no significant effect of sex on the attributes of lightness (L*), redness (a*), and yellowness (b*) of goose meat. In contrast, Uhliřová et al. (2018) showed significantly higher redness (a*) in the breast muscles of 8- and 16-wk-old male Eskildsen Schwer and Czech Goose geese compared to the breast muscles of females of the same age. This study found significantly lower lightness (L*) and higher redness (a*) values in breast muscles compared to leg muscles, indicating a darker color of breast muscles. This was probably related to differences in the percentage of oxidative fibers and the concentration of myoglobin and hemoglobin. In a study by Gumułka et al. (2009), the *m. pectoralis superficialis* (breast muscle) of 17-wk-old White Kołuda geese contained 73.1% oxidative – red fibers and 25.7% glycolyte – white fiber. In the case of m. *biceps femoris* (leg muscle), the respective values were 24.9% − red fiber and 49.2% − white fiber. The significantly higher iron content in breast muscles than in leg muscles resulted in a higher red color saturation (a*), which may indicate a higher concentration of myoglobin and hemoglobin in the meat, which contain iron. These results are consistent with the results of studies conducted on Turkish geese (Oz and Celik, 2015).

The sex of the birds strongly influenced the intensity of the aroma of the breast muscles, which was significantly (*P* = 0.012) greater in females than in males. It was also found that the breast muscles were characterized by a significantly lower intensity of aroma (*P* < 0.001) and aroma desirability (*P* < 0.001), tastiness intensity (*P* = 0.017), and juiciness (*P* < 0.001) compared to leg muscles (Table 7). Uhliřová et al. (2018) report a significantly higher flavor intensity of breast muscles in males than in females of 16-wk-old Eskildsen Schwer and Czech Goose geese, which was probably due to the higher fat content in the breast muscles of males than in females. In turn, Lisiak et al. (2021) found slightly higher flavor, juiciness, and tenderness scores for breast muscles of 17-wk-old Zatorska geese than for 15-week-old White Kołuda® geese in this study, using the same 5-point sensory evaluation scale. Based on the analysis of the results of this study (Table 8), it was also found that the pectoral muscles of 15 -wk-old male geese were significantly less tender (higher maximum shear force), harder, more elastic, chewier, and gummy (*P* = 0.001 to 0.044), and had a higher sum of elastic moduli (*P* = 0.022) compared to the muscles of females of the same age. The texture characteristics (WB shear force, hardness, cohesiveness, springiness, chewiness, gumminess) of the *pectoralis major* muscle after heat treatment of 15-wk-old White Kołuda® geese obtained in this study were lower than those assessed by Kokoszyński et al. (2022) conducted on the pectoralis major muscle of geese from genetic resource flocks (Landes, Slovak White, Kuban) after four reproductive seasons, which was probably related to the smaller diameter of their muscle fibers and the thickness of connective tissue (*perimysium, endomysium*) in younger birds (Gardzielewska et al., 2009). Haraf et al. (2023), conducting research on 17-wk-old female geese of four Polish native varieties, recorded higher hardness and gumminess, but lower cohesiveness, springiness, chewiness, and WB shear force of the *pectoralis major* muscle compared to the breast muscle of 15-wk-old female White Kołuda® geese in this study (Table 2).

**Table 2:** The weight of edible offal of male and female White Kołuda® geese at 15 weeks of age.

Item	Heart (g)	Gizzard (g)	Liver (g)	Feet (g)
Male	40.97^a^ ± 4.44	221.14 ± 30.44	107.38^a^ ± 16.17	172.24^a^ ± 10.37
Female	34.34^b^ ± 4.19	199.37 ± 33.11	89.85^b^ ± 18.69	144.9^b^ ± 15.39
Average	37.65 ± 5.20	210.26 ± 33.36	98.61 ± 19.99	158.61 ± 17.52
SEM	0.92	5.90	3.54	3.10
*P* value	<0.001	0.064	0.010	<0.001

Note: Values are expressed as means ± standard deviation, *n* = 16 for gender.

^a,b^*P* < 0.05 – means with different superscripts are statistically different between male and female.

SEM - standard error of the mean.

**Table 3:** Basic chemical composition of breast muscle or thigh muscle of male and female White Kołuda® geese at the age of 15 weeks.

Item		Water (%)	Intramuscular fat (%)	Protein (%)	Collagen (%)
Male	BM	69.63 ± 0.35	4.03^a^ ± 0.28	21.32^b^ ± 0.44	1.21 ± 0.07
	TM	70.04 ± 0.78	5.19^b^ ± 0.68	20.97 ± 0.37	1.55^a^ ± 0.16
Female	BM	70.00 ± 0.45	3.66^b^ ± 0.70	22.32^a^ ± 0.43	1.19 ± 0.06
	TM	69.51 ± 0.78	6.33^a^ ± 0.65	20.98 ± 0.32	1.50^b^ ± 0.14
Average	BM	69.82 ± 0.44	3.84 ± ± 0.56	21.82* ± 0.66	1.20 ± 0.07
	TM	69.77 ± 0.81	5.76* ± 0.87	20.98 ± 0.34	1.53* ± 0.05
SEM		0.14	0.15	0.06	0.01
*P* value					
Gender (G)		0.609	0.014	<0.001	<0.001
Type of muscle (M)		0.793	<0.001	<0.001	<0.001
Interaction G x M		0.006	<0.001	<0.001	0.736

Note: Values are expressed as means ± standard deviation, *n* = 16 for gender, *n* = 32 for type of muscle.

^a,b^*P* < 0.05 – means with different superscripts are statistically different between male and female.

⁎ are statistically different between the breast muscle and thigh muscle in both sexes of birds. BM - breast muscle, TM - thigh muscle, SEM - standard error of the mean.

**Table 4:** Contents of selected macroelements of breast muscle or thigh muscle of male and female White Kołuda® geese at 15 weeks of age.

Item		Contents of macroelements (mg/100 g of meat)				
		K - potassium	P - phosphorus	Na - sodium	Mg - magnesium	Ca - calcium
Male	BM	297.51^b^ ± 28.44	213.77^b^ ± 14.46	49.00 ± 7.35	24.80 ± 3.63	3.38 ± 0.43
	TM	283.51^b^ ± 42.05	196.14^b^ ± 28.84	67.77 ± 13.89	25.13 ± 6.15	3.28 ± 0.78
Female	BM	310.06^a^ ± 21.00	230.25^a^ ± 11.18	45.73 ± 5.56	25.94 ± 1.90	3.03 ± 0.41
	TM	306.94^a^ ± 39.17	217.76^a^ ± 23.69	67.08 ± 12.90	26.30 ± 3.49	3.22 ± 0.71
Average	BM	303.78 ± 27.13	222.01* ± 15.23	47.36 ± 6.63	25.37 ± 2.91	3.21 ± 0.45
	TM	295.22 ± 41.71	206.95 ± 28.19	67.43* ± 13.19	25.71 ± 4.96	3.25 ± 0.73
SEM		4.39	2.98	1.82	0.51	0.07
*P* value						
Gender (G)		0.008	0.001	0.455	0.266	0.180
Type of muscle (M)		0.674	0.005	<0.001	0.737	0.773
Interaction G x M		0.959	0.623	0.626	0.988	0.329

Note: Values are expressed as means ± standard deviation, *n* = 16 for gender, *n* = 32 for type of muscle.

^a,b^*P* < 0.05 – means with different superscripts are statistically different between male and female.

⁎ are statistically different between the breast muscle and thigh muscle in both sexes of birds. BM - breast muscle, TM - thigh muscle, SEM - standard error of the mean.

**Table 5:** Contents of selected microelements of breast muscle or thigh muscle of male and female of White Kołuda® geese at 15 weeks of age.

Item		Contents of microelements (mg/100 g of meat)				
		Fe - iron	Zn - zinc	Cu - copper	Mn - manganese	Cr - chrome
Male	BM	2.35 ± 0.77	1.31^a^ ± 0.28	0.31 ± 0.09	0.03 ± 0.01	0.03 ± 0.01
	TM	1.48 ± 0.25	2.90^a^ ± 0.63	0.12 ± 0.04	0.04 ± 0.01	0.06 ± 0.02
Female	BM	2.54 ± 0.87	1.04^b^ ± 0.19	0.29 ± 0.07	0.03 ± 0.01	0.03 ± 0.01
	TM	1.58 ± 0.34	2.54^b^ ± 0.74	0.12 ± 0.03	0.04 ± 0.01	0.06 ± 0.02
Average	BM	2.45* ± 0.82	1.18 ± 0.27	0.30* ± 0.08	0.03 ± 0.01	0.03 ± 0.01
	TM	1.53 ± 0.30	2.72* ± 0.70	0.12 ± 0.03	0.04* ± 0.01	0.06* ± 0.02
SEM		0.09	0.12	0.01	0.01	0.01
*P* value						
Gender (G)		0.362	0.017	0.413	0.376	0.481
Type of muscle (M)		<0.001	<0.001	<0.001	0.007	<0.001
Interaction G x M		0.755	0.738	0.443	0.351	0.798

Note: Values are expressed as means ± standard deviation, *n* = 16 for gender, *n* = 32 for type of muscle.

^a,b^*P* < 0.05 – means with different superscripts are statistically different between male and female.

⁎ are statistically different between the breast muscle and thigh muscle in both sexes of birds. BM - breast muscle, TM - thigh muscle, SEM - standard error of the mean.

**Table 6:** Physicochemical properties of breast muscle or thigh muscle of male and female White Kołuda® geese at 15 weeks of age.

Item		Acidity (pH_24_)	Electrical conductivity (mS/cm)	Termal loss (%)	Lightness (L*)	Redness (a*)	Yellowness (b*)
Male	BM	6.18 ± 0.10	8.83 ± 1.59	30.47^a^ ± 3.32	36.88 ± 4.38	14.34 ± 2.70	2.28^a^ ± 1.55
	TM	6.24 ± 0.11	8.29 ± 2.12	31.29^a^ ± 2.19	41.83 ± 3.92	11.62 ± 3.46	1.68^a^ ± 2.46
Female	BM	6.15 ± 0.10	9.93 ± 1.35	27.68^b^ ± 2.13	38.42 ± 4.70	13.31 ± 2.21	−0.09^b^ ± 2.02
	TM	6.32 ± 0.07	6.48 ± 1.54	27.65^b^ ± 3.14	41.78 ± 4.64	10.88 ± 2.71	0.05^b^ ± 1.53
Average	BM	6.17 ± 0.10	9.38* ± 1.56	29.07 ± 3.09	37.65 ± 4.54	13.82* ± 2.48	1.09 ± 2.14
	TM	6.28* ± 0.10	7.38 ± 2.04	29.47 ± 3.24	41.81* ± 4.23	11.52 ± 3.08	0.88 ± 2.18
SEM		0.02	0.36	0.57	0.75	0.55	0.39
*P* value							
Gender (G)		0.351	0.398	<0.001	0.502	0.212	0.001
Type of muscle (M)		<0.001	<0.001	0.567	0.001	0.001	0.655
Interaction G x M		0.022	0.001	0.535	0.476	0.843	0.441

Note: Values are expressed as means ± standard deviation, *n* = 16 for gender, *n* = 32 for type of muscle.

^a,b^*P* < 0.05 – means with different superscripts are statistically different between male and female.

⁎ are statistically different between the breast muscle and thigh muscle in both sexes of birds. BM - breast muscle, TM - thigh muscle, SEM - standard error of the mean.

**Table 7:** Sensoric properties of breast muscle or thigh muscle of male and female White Kołuda® geese at 15 weeks of age.

Item		Aroma intensity (pts.)	Aroma desirability (pts.)	Juiciness (pts.)	Tenderness (pts.)	Tastiness intensity (pts.)	Tastiness desirability (pts.)
Male	BM	3.59^b^ ± 0.30	3.73 ± 0.39	3.44 ± 0.35	3.83 ± 0.33	4.13 ± 0.32	4.09 ± 0.20
	TM	4.20 ± 0.26	4.25 ± 0.27	3.92 ± .22	3.61 ± 0.29	4.39 ± .0.41	3.83 ± 0.28
Female	BM	4.03^a^ ± 0.34	3.83 ± 0.25	3.63 ± 0.29	3.72 ± 0.31	4.11 ± 0.38	4.05 ± 0.41
	TM	4.14 ± 0.24	3.95 ± 0.25	3.97 ± 0.41	3.66 ± 0.34	4.28 ± .38	4.19 ± 0.35
Average	BM	3.81 ± 0.39	3.78 ± 0.33	3.53 ± 0.33	3.77 ± 0.32	4.12 ± 0.31	4.07 ± 0.32
	TM	4.17* ± 0.25	4.10* ± 0.30	3.95* ± 0.32	3.64 ± 0.31	4.34* ± 0.39	4.01 ± 0.36
SEM		0.04	0.05	0.06	0.05	0.07	0.06
*P* value							
Gender (G)		0.012	0.177	0.152	0.329	0.485	0.056
Type of muscle (M)		<0.001	<0.001	<0.001	0.051	0.017	0.439
Interaction G x M		0.001	0.011	0.387	0.695	0.606	0.014

Note: Values are expressed as means ± standard deviation, *n* = 16 for gender, *n* = 32 for type of muscle.

^a,b^*P* < 0.05 – means with different superscripts are statistically different between male and female.

⁎ are statistically different between the breast muscle and thigh muscle in both sexes of birds. BM - breast muscle, TM - thigh muscle, SEM - standard error of the mean, pts. – points.

**Table 8:** Texture and rheological properties of the pectoralis major muscle of male and female White Kołuda® geese at 15 weeks of age.

Item	WB shear force (N)	Hardnes (N)	Cohesiveness	Springiness	Chewiness (N)	Gumminess (N)	Sum of elastic moduli (kPA)	Sum of viscous moduli (kPa x s)
Male	63.77 ± 7.60	43.66^a^ ± 3.41	0.45 ± 0.03	1.48^a^ ± 0.05	29.15^a^ ± 3.26	19.68^a^ ± 2.14	409.86^a^ ± 92.92	14407.16 ± 4092.98
Female	55.70 ± 10.90	39.35^b^ ± 3.69	0.43 ± 0.03	1.45^b^ ± 0.05	24.99^b^ ± 3.46	17.23^b^ ± 2.13	342.43^b^ ± 85.62	12907.88 ± 4092.98
Average	59.73 ± 0.35	41.50 ± 4.14	0.44 ± 0.03	1.46 ± 0.05	27.07 ± 4.02	18.45 ± 2.51	376.15 ± 84.65	13657.52 ± 38955.05
SEM	0.06	0.73	0.01	0.01	0.71	0.44	14.98	6895.69
P value	0.025	0.001	0.174	0.044	0.002	0.004	0.022	0.278

Note: Values are expressed as means ± standard deviation, *n* = 16/gender.

^a,b^*P* < 0.05 – means with different superscripts are statistically different between male and female.

SEM - standard error of the mean.

Collagen, as the main component of connective tissue, significantly shapes the viscoelastic properties of meat, and its higher content leads to an increased elastic modulus. A positive correlation has been demonstrated between collagen content and texture parameters and elastic modulus in poultry meat, resulting from its role as a structural reinforcement of muscle tissue (Liu et al., 1995; Purslow, 2005). At the same time, the degree of collagen cross-linking limits stress relaxation and increases resistance to deformation (Nishimura, 2010). Goose meat, characterized by a relatively higher connective tissue content compared to chicken meat, exhibits higher elastic moduli, which may result in slightly higher hardness and reduced susceptibility to deformation (Wołoszyn et al., 2020).

## Conclusions

In summary, it can be concluded that male carcasses were characterized by a slightly higher total content of breast and leg muscles and a higher proportion of less valuable parts of the carcass, i.e., wings, neck, and fat. The breast muscles of males were harder, more rubbery, and less tender. In addition, it was found that the breast muscles of White Kołuda® geese have a higher protein content and less intramuscular fat and collagen, as well as a higher concentration of phosphorus, iron, and copper, but lower sodium, zinc, manganese, and chromium content compared to leg muscles. This may indicate that male carcasses and meat are less suitable for consumers and the processing industry compared to female carcasses and meat, and that breast meat has a higher nutritional value than leg meat. This study is one of the few studies examining textural characteristics and mineral concentration. The results of this study supplement the current state of knowledge with information on the electrical conductivity and rheological properties of White Kołuda®. goose meat.

## CRediT authorship contribution statement

**Dariusz Kokoszyński:** Writing – review & editing, Writing – original draft, Supervision, Methodology, Investigation, Formal analysis, Data curation, Conceptualization. **Dorota Cygan-Szczegielniak:** Writing – review & editing, Methodology, Investigation. **Arkadiusz Nędzarek:** Writing – review & editing, Methodology, Investigation. **Hanna Jankowiak:** Methodology, Investigation.

## Disclosures

The authors hereby confirm that the manuscript has not been published in whole or in part, nor is being considered for publication elsewhere and all authors are in agreement with the content of the manuscript. The authors declare any financial support from or relationships that may pose conflict of interest.
